# Human adipose tissue-derived small extracellular vesicles promote soft tissue repair through modulating M1-to-M2 polarization of macrophages

**DOI:** 10.1186/s13287-023-03306-7

**Published:** 2023-04-07

**Authors:** Jia Dong, Bin Wu, Weidong Tian

**Affiliations:** 1 State Key Laboratory of Oral Disease and National Clinical Research Center for Oral Diseases and National Engineering Laboratory for Oral Regenerative Medicine, West China School of Stomatology, Sichuan University, Sichuan 610041 Chengdu, China; 2 Department of Stomatology, People’s Hospital of Longhua Shenzhen, Shenzhen, 518109 Guangdong China

**Keywords:** Human adipose tissue-derived small extracellular vesicles, Adipose tissue regeneration, Skin wound healing, Macrophage polarization

## Abstract

**Background:**

Successful regenerative medicine strategies need the manipulation and control of macrophages’ phenotypic switching. Our previous study indicated that rat and porcine adipose tissue-derived small extracellular vesicles could successfully promote soft tissue repair. However, whether human adipose tissue-derived small extracellular vesicles (h-sEV-AT) showed the same ability to promote soft tissue regeneration and whether adipose tissue-derived small extracellular vesicles (sEV-AT) contribute to modulating the polarization of macrophages were unknown.

**Methods:**

In this study, we, for the first time, isolated h-sEV-AT from liposuction adipose tissue and characterized the morphology, size distribution, and marker protein. In vitro, we treated adipose-derived stromal/stem cells (ASCs), endothelial cells (ECs), and M1 macrophages with h-sEV-AT. In vivo, the ability of h-sEV-AT to promote soft tissue regeneration and polarize macrophages was investigated.

**Results:**

The results indicated that h-sEV-AT possessed the characteristics of small extracellular vesicles (sEVs). In vitro, an obvious increase in adipogenesis and angiogenesis was induced by h-sEV-AT. In vivo, h-sEV-AT successfully induced the regeneration of adipose tissue and effectively accelerated full-thickness skin wound healing. Besides, we found that h-sEV-AT showed the ability to increase the percentage of M2 macrophages both in vivo and in vitro, which had been reported to contribute to tissue repair and regeneration.

**Conclusions:**

Taken together, these results suggested that h-sEV-AT showed the ability to induce soft tissue repair supported by not only the differentiation of ASCs and ECs but also the polarization of macrophages. Considering the abundant sources, high yield, and guaranteed effectiveness, this study provided a cell-free strategy for soft tissue regeneration that directly isolated small extracellular vesicles from human liposuction adipose tissue.

**Supplementary Information:**

The online version contains supplementary material available at 10.1186/s13287-023-03306-7.

## Background

Small extracellular vesicles (sEVs), released from different cells and tissues, were considered a new additive biomaterial through the transport of multiple proteins, nucleic acids, and lipids [1–5]. They have been extensively studied in various fields as therapeutic targets, diagnosis tools, drug delivery vectors, and inducers of tissue regeneration [4]. For soft tissue regeneration, sEVs have been reported to recruit host cells, regulate immune responses, stimulate angiogenesis, and promote tissue repair or regeneration [6–8].

Cell-free induced tissue regeneration began with immediate adsorption of a large number of host proteins to the surface of the implants, followed by rapid neutrophil infiltration and activation. Then, tissue-surrounding macrophages and circulating monocytes entered the implants to engulf and digest the foreign body [9]. All these host reactions made up the immune response that evoked the regeneration process. If the immune response was well controlled, the regenerated tissue will be architecture-morphological equivalency to normal tissue. However, if the immune response was uncontrolled and dysregulated, the host response always ended with chronic inflammation, fibrosis, and even necrosis of tissues [10]. With all stages, macrophages exhibit extraordinary plasticity [11].

Macrophage phenotypes were generally defined as pro-inflammatory M1 macrophages and anti-inflammatory M2 macrophages, respectively [12]. M1 macrophages promoted inflammatory response with intensive releasing of pro-inflammatory factors, like interleukin 6 (IL-6), interleukin 1 beta (IL-1β), and tumor necrosis factor-alpha (TNF-α). In contrast, M2 macrophages antagonized M1 macrophages’ responses and reduced inflammatory response by releasing anti-inflammatory factors, like interleukin 4 (IL-4), interleukin 13 (IL-13), transforming growth factor-beta 1 (TGF-β1), and interleukin 10 (IL-10), functioning in tissue regeneration and remodeling [11, 12].

Our previous study has shown that rat and porcine adipose tissue-derived small extracellular vesicles effectively promoted soft tissue regeneration [13–16]. However, whether small extracellular vesicles could be isolated from human liposuction adipose tissue and whether it also showed effectiveness on soft tissue were unknown. Besides, the role of sEV-AT in guiding macrophage polarization was elusive.

In this study, we, for the first time, isolated h-sEV-AT from liposuction adipose tissue and investigated the differentiation of ASCs and ECs induced by h-sEV-AT. In vivo, the effect of h-sEV-AT on rat adipose tissue regeneration and full-thickness skin wound were evaluated. Besides, the polarization of macrophages induced by adipose tissue-derived small extracellular vesicles was also investigated here.

## Methods

### h-sEV-AT isolation and characterization

Subcutaneous adipose tissue was collected from the abdomen of four healthy females (age, 25 ± 5 years, body mass index [BMI]: 19–22) after obtaining their consent. All participants agreed to offer their liposuction tissue and were informed of the procedures and purpose of the study. This study was approved by the Ethics Committees of the State Key Laboratory of Oral Diseases, West China School of Stomatology, Sichuan University. The approval number was WCHSIRB-CT-2022–281. After washing with phosphate-buffered saline (PBS), adipose tissue was cut into small pieces and transferred into a flask to culture for 2 days. Then, the supernatant was collected, filtered, and concentrated. The concentrated extract medium was mixed with 0.5 volume of Total Exosome Isolation™ reagent (Life Technologies, USA), incubated overnight at 4 °C, and spun down at 10,000 g, 4 °C for 1 h. The pellet was resuspended in PBS for further experiments.

The isolated h-sEV-AT was visualized using a transmission electron microscope (TEM, USA) by negative staining. The particle size and size distribution of h-sEV-AT were determined by Zeta View analysis system (Particle Metrix, Germany) according to the manufacturer’s protocol. The marker proteins were detected by Western blot according to our previous methods [13, 16]. Briefly, 30 μg h-sEV-AT mixed with loading buffer (Solarbio, China), boiled for 10 min, resolved on 10% polyacrylamide gel, and blotted onto a nitrocellulose membrane. Primary antibodies, CD81(1:1000, Zen Bioscience, 381296), TSG101(1:1000, Abcam, Ab125011), CD9 (1:1000, Zen Bioscience, 380411), CD63 (1:1000, Zen Bioscience, R23327), GM130(1:1000, Abcam, ab52649), and Actin (1:5000, Abcam, ab179467), were used and followed by Horseradish peroxidase (HRP) conjugated secondary antibodies. High sig ECL Western Blotting Substrate (Tanon, China) was used for detecting protein signals by ImageQuant LAS 4000 mini machine (GE Healthcare, USA).

### h-sEV-AT labeling and cellular uptake

ASCs and ECs were prepared and cultured exactly as described previously [13]. For cellular uptake, 100 μg h-sEV-AT, dissolved in 1 ml culture medium, were labeled with 1 μg membrane-labeling dye DiO (Life Tech, V22886) for 30 min, re-purified with the Total Exosome Isolation™ reagent (Life Technologies, USA) to remove the unincorporated dye. The obtained DiO-labeled h-sEV-AT pellet was cultured with ASCs or ECs for 6 h, respectively. These labeled cells were washed with PBS, fixed in 4% paraformaldehyde, stained with phalloidin (1:200, Invitrogen, A34055-300U) and DAPI (1:1000, Solarbio, C0050), washed with PBS, and imaged by confocal microscopy (Olympus FV1000, Japan).

### Cell differentiation induced by h-sEV-AT

ASCs were seeded into 24-well plates with a density of 1 × 10^5^ per well, and continuously cultured with 0.5 ml culture medium containing 50 μg h-sEV-AT. The culture medium only was considered as the blank group. The culture medium was changed every 2 days to maintain the treatment effect. After culturing ASCs with h-sEV-AT for 10 days, the relative expression of adipogenic genes (PPARγ, C/EBPα, adiponectin, and FABP4) was analyzed by real-time PCR and primer sequences are listed in Table 1 (*n* = 3). After culturing for 16 days, lipid clusters were stained with oil red O and imaged by an inverted microscope (Olympus DP80, Japan) (*n* = 3).

**Table 1:** Primers for qRT-PCR

Target cDNA	Primer sequence (5′–3′)
Rat-GAPDH	TATGACTCTACCCACGGCAAG TACTCAGCACCAGCATCACC
Rat-PPARγ2	GCCCTTTGGTGACTTTATGGAG GCAGCAGGTTGTCTTGGATGT
Rat-C/EBPα	GCCAAGAAGTCGGTGGATAAGA GTCACTGGTCAACTCCAACACCT
Rat-Adiponectin	CGTTCTCTTCACCTACGACCAGT ATTGTTGTCCCCTTCCCCATAC
Rat-FABP4	GTAGAAGGGGACTTGGTCGTCAT ACTTTCCTGTCATCTGGGGTGA
Rat-CD31	CCAGGTGCTATTCTATAAGGACGATGC TGGAAGACCCGAGACTGAGGAATG
Rat-VEGF	GGAGAGGAGCCCGCCAAGG GCAGTAAAGCCAGGGTCCAGTG
Rat-FGF2	GAGCGACCCACACGTCAAACTAC CAGCCGTCCATCTTCCTTCATAGC
Rat-Angiogenin	GATGAGCCTGCGTCCTCTGTTG ATCTGGCATCCCGACCCTTGG
Mouse-GAPDH	AAGAAGGTGGTGAAGCAGGCATC CGGCATCGAAGGTGGAAGAGTG
Mouse-IL-6	CTCTGGGAAATCGTGGAAAT CCAGTTTGGTAGCATCCATC
Mouse-IL-1β	ATCTCGCAGCAGCACATCAA ATGGGAACGTCACACACCAG
Mouse-TNF-α	GTGCCAGCCGATGGGTTGTAC TGACGGCAGAGAGGAGGTTGAC
Mouse-iNOS	CGGACGAGACGGATAGGCAGAG GGAAGGCAGCGGGCACATG
Mouse-Arg1	AGTGTGGTGCTGGGTGGAGAC GCTGGTTGTCAGGGGAGTGTTG
Mouse-TGF-β1	ATGGTGGACCGCAACAACGC GGCACTGCTTCCCGAATGTCTG
Mouse-IL-10	GAGGATCAGCAGGGGCCAGTAC AAGGCAGTCCGCAGCTCTAGG
Mouse-CD206	TCAATGCCACTGCCATGCCTAC AGCTTGCCGTGCGTCTTGC

ECs were seeded into 24-well plates with a density of 1 × 10^5^ per well and continuously cultured with 0.5 ml culture medium containing 50 μg h-sEV-AT. The culture medium only was considered as the blank group. The culture medium was changed every 2 days to maintain the treatment effect. After culturing ECs with h-sEV-AT for 4 days, the relative expression of angiogenic genes (CD31, VEGF, FGF2, and angiogenin) was determined by real-time PCR and primer sequences are listed in Table 1 (*n* = 3). For tube formation, ECs were suspended in the culture medium with h-sEV-AT and seeded into 96-well plates coated with Matrigel (Corning, USA). After incubation for 5 h, images were acquired on an inverted microscope (Olympus DP80, Japan) (*n* = 3).

### Macrophage polarization induced by h-sEV-AT

M0 macrophages (the primary Raw 264.7 cell line) were seeded at 1 × 10^5^ cells per well into 24-well plates and 100 ng/ml LPS (Sigma) was added to induce M1 macrophages. Then, M1 macrophages were continuously cultured with 0.5 ml culture medium containing 50 μg h-sEV-AT. The culture medium only was considered as the blank group. The culture medium was changed every 2 days to maintain the treatment effect. After 4 days of induction, the percentage of M1 macrophages and M2 macrophages was analyzed by immunofluorescence staining (*n* = 3). The relative expression of M1 marker genes (IL-6, IL-1β, TNFα, and iNOS) and M2 marker genes (Arg1, TGF-β1, IL-10, and CD206) was analyzed by qRT-PCR, and primer sequences are listed in Table 1 (*n* = 3).

### Animal experiments

4-week-old male SD rats (75 ± 10 g, *n* = 12) were obtained from Dashuo Experimental Animal Co. Ltd. (Chengdu, China). All operations of animals were reviewed and approved by the Ethics Committees of the State Key Laboratory of Oral Diseases, West China School of Stomatology, Sichuan University. The approval number was WCHSIRB-D-2020–391.

In the adipose tissue regeneration experiments, 4-week-old male SD rats (75 ± 10 g, *n* = 4) were under general anesthesia with 1% pentobarbital sodium (10 ml/kg, intraperitoneal injection). Two custom-designed silicone tubes (diameter: 4.87 mm, height: 5 mm) were subcutaneously implanted into the back on each side, which contained 120 μl Matrigel only or 120 μl Matrigel mixed with 720 μg h-sEV-AT. Rats are raised separately. All rats (*n* = 4) were euthanized via rapid cervical dislocation at 4 weeks, and the samples were harvested for further analysis. No animal was excluded.

In the full-thickness skin wound healing experiments, 4-week-old male SD rats (75 ± 10 g, *n* = 8) were under general anesthesia by 1% pentobarbital sodium (10 ml/kg, intraperitoneal injection). Two full-thickness skin wounds (20 mm in diameter) were made on each side. 100 μl PBS only or 100 μl PBS containing 600 μg h-sEV-AT were subcutaneously injected around the wounds at 4 points. Rats are raised separately. To ensure the therapeutic effect of sEV-AT, injections were performed once a week. Digital photographs were taken every 4 days until the wounds in one group were completely healed. The wound area between the two groups was significantly different on day 6, so digital photographs at this time point were taken. The wound area was measured using the Image J software. Rats were euthanized on day 8 and day 16, respectively (*n* = 4 per timepoint), via rapid cervical dislocation, and the samples were harvested for further testing. No animal was excluded.

### Hematoxylin and eosin (H&E) staining

The samples were harvested and fixed in 4% neutral paraformaldehyde overnight, dehydrated, paraffin-embedded, and sectioned into 5–6 µm thick sections for H&E staining, Masson staining, immunochemical staining, and immunofluorescence staining.

### Immunochemical staining

Sections were blocked for 2 h and then incubated overnight at 4 °C with primary antibodies. Primary antibodies against perilipin A (1:200, Abcam, ab3526) and CD31(1:200, Santa Cruz, sc-376764) were used in this study for analyzing adipocytes and blood vessels. The secondary antibody was shown by the DAB kit (Gene Tech, Shanghai, China). Images were captured by an inverted microscope (Olympus DP80, Japan) (*n* = 3).

### Immunofluorescence staining

Cells or sections were blocked for 2 h and then incubated overnight at 4 °C with primary antibodies. Primary antibodies against F4/80 (1:200, Abcam, ab240946) and iNOS (1:200, Santa Cruz, sc-7271) or CD206 (1:200, Santa Cruz, sc-58986) were used in this study to analyze M1 and M2 macrophages. The secondary antibodies goat anti-mouse 488 (1:200, Invitrogen, A11008) and goat anti-rabbit 555 (1:200, Invitrogen, A21428) were followed. DAPI (1:1000, Solarbio, C0050) was used to mark the nuclei. Images were captured by confocal microscopy (Olympus FV1000, Japan) using the same imaging threshold and exposure time for each experiment. The number of positive cells per field was calculated with ImageJ software. The results were further analyzed using GraphPad Prism 7 software (*n* = 3).

### Statistical analysis

All statistical analyses were performed using Microsoft Excel or GraphPad Prism 7 software. All results were expressed as mean value ± standard deviation. An unpaired two-tailed Student’s *t* test was used to determine the level of significance. *p < 0.05, **p < 0.01, ***p < 0.001, ****p < 0.0001.

## Results

### Characterization of h-sEV-AT

**Fig. 1:**
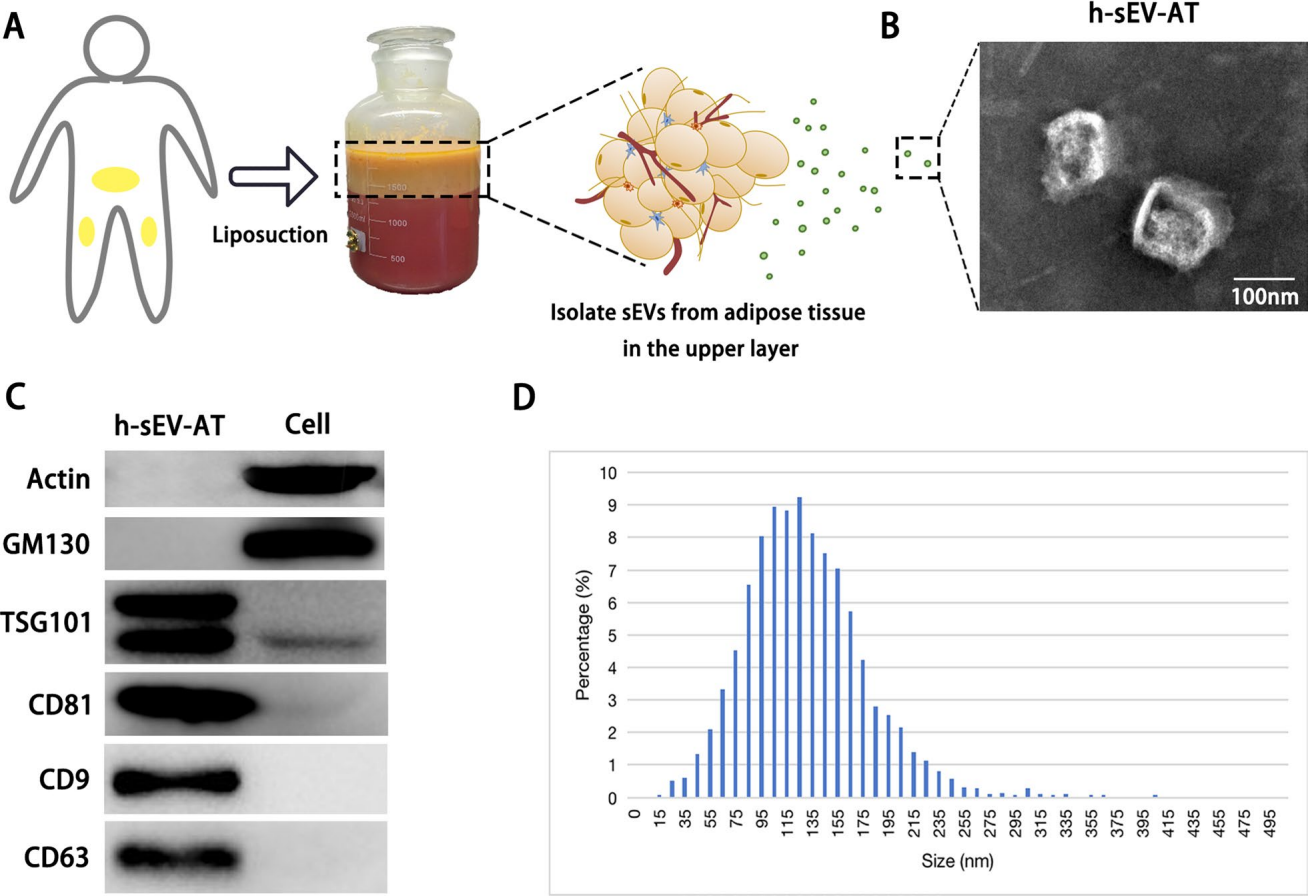
Characterization of h-sEV-AT. **a** Schematic view of the process of isolating h-sEV-AT from human liposuction adipose tissue. **b** Representative image of h-sEV-AT with transmission electron microscopy. Scale bar = 100 nm. **c** Western blot analysis of exosomal proteins, CD81, TSG101, CD9, and CD63. Actin and GM130 were cellular proteins as control. All full-length blots were presented in Additional file 1: Figure S1. **d** The particle size distribution of sEV-AT was measured by ZataView analysis

Human adipose tissue-derived small extracellular vesicles (h-sEV-AT) were isolated from liposuction adipose tissue (Fig. 1A). The morphology of h-sEV-AT was analyzed by transmission electron microscopy (TEM) and the results showed that h-sEV-AT displayed a round shape or a double-membrane spherical structure (Fig. 1B). Western blot analysis confirmed that h-sEV-AT expressed the exosomal proteins (CD81, TSG101, CD9, CD63), and not expressed the cellular proteins (actin and GM130) (Fig. 1C and Additional file 1: Figure S1). Nanoparticle tracking analysis (NTA) was used for evaluating the size distribution of h-sEV-AT. The diameter of h-sEV-AT was mainly between 25 and 240 nm and showed certainly homogenous. The peak was 120 nm (Fig. 1D). These results indicated that h-sEV-AT possessed the characteristics of sEVs.

### The differentiation of ASCs and ECs induced by h-sEV-AT

**Fig. 2:**
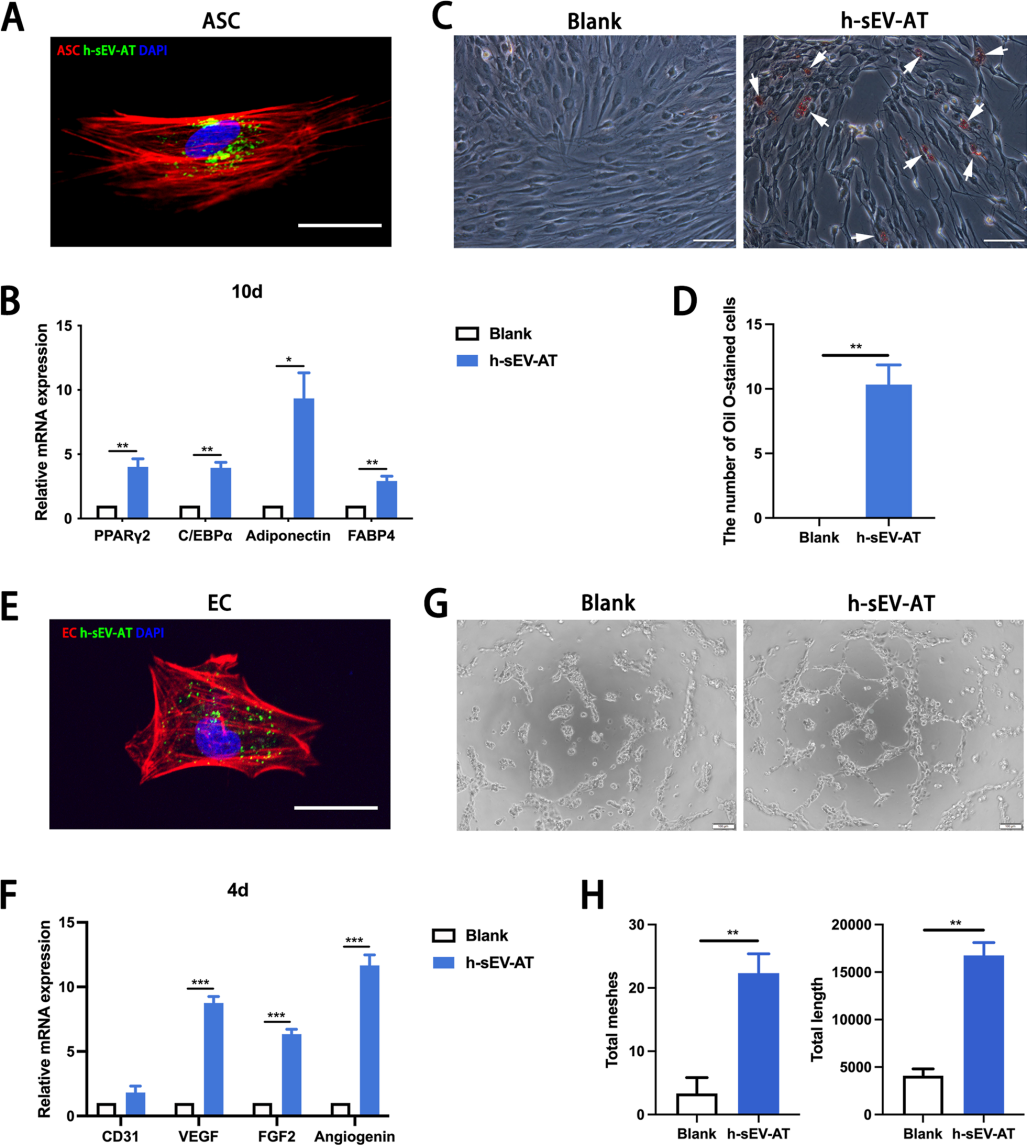
Cell differentiation induced by h-sEV-AT. **a** Uptake analysis of h-sEV-AT by ASCs (red: phalloidin-stained ASC, green: Dio-labeled h-sEV-AT, blue: DAPI-stained nuclei). Scale bar = 10 µm. **b** After culturing ASCs with h-sEV-AT for 10 days, the relative expression of adipogenic marker genes (PPARγ2, C/EBPα, adiponectin, and FABP4) was measured by real-time PCR (*n* = 3). **c** Representative images of Oil Red O-stained cells after culturing ASCs with h-sEV-AT for 16 days. The white arrows pointed out positive-stained cells. Scale bar = 200 µm. **d** The number of Oil Red O-stained cells per field of view (scale bar = 200 µm) was analyzed (*n* = 3). **e** Uptake analysis of h-sEV-AT by ECs (red: phalloidin-stained EC, green: Dio-labeled h-sEV-AT, blue: DAPI-stained nuclei). Scale bar = 10 µm. **f** After culturing ECs with h-sEV-AT for 4 days, the relative expression of angiogenic marker genes (CD31, VEGF, FGF2, and angiogenin) was measured by real-time PCR (*n* = 3). **g** Representative images of tube-like structures after culturing ECs with h-sEV-AT for 5 h. Scale bar = 200 µm. **h** Total meshes and total length per field of view (scale bar = 200 µm) were analyzed by ImageJ software (*n* = 3). The significance was tested with an unpaired two-tailed Student’s *t* test. *p < 0.05, **p < 0.01, ***p < 0.001

To investigate the effect of h-sEV-AT on the differentiation of ASCs and ECs, we first evaluated the cellular uptake of h-sEV-AT by these cells. The results showed that h-sEV-AT could be taken by ASCs (Fig. 2A). After culturing ASCs with h-sEV-AT for 10 days, the expression of adipogenic marker genes (PPARγ2, C/EBPα, adiponectin, and FABP4) was upregulated (Fig. 2B). After culturing for 16 days, there were lipid droplets accumulated in ASCs stained by Oil Red O in the h-sEV-AT group. However, in the blank group, there were no Oil Red O-stained cells (Fig. 2C-2D). Similarly, for angiogenesis, h-sEV-AT could be taken by ECs (Fig. 2E). After culturing ECs with h-sEV-AT for 4 days, the expression of angiogenesis marker genes (CD31, VEGF, FGF2, and angiogenin) was also upregulated by h-sEV-AT compared with that of the blank group (Fig. 2F). Besides, h-sEV-AT could also promote tube-like structures formation in ECs (Fig. 2G-2H). Taken together, h-sEV-AT showed the ability to promote the differentiation of ASCs or ECs, which was similar to that of rat and porcine-derived sEV-AT in our previous study [13].

### Macrophage polarization induced by h-sEV-AT

**Fig. 3:**
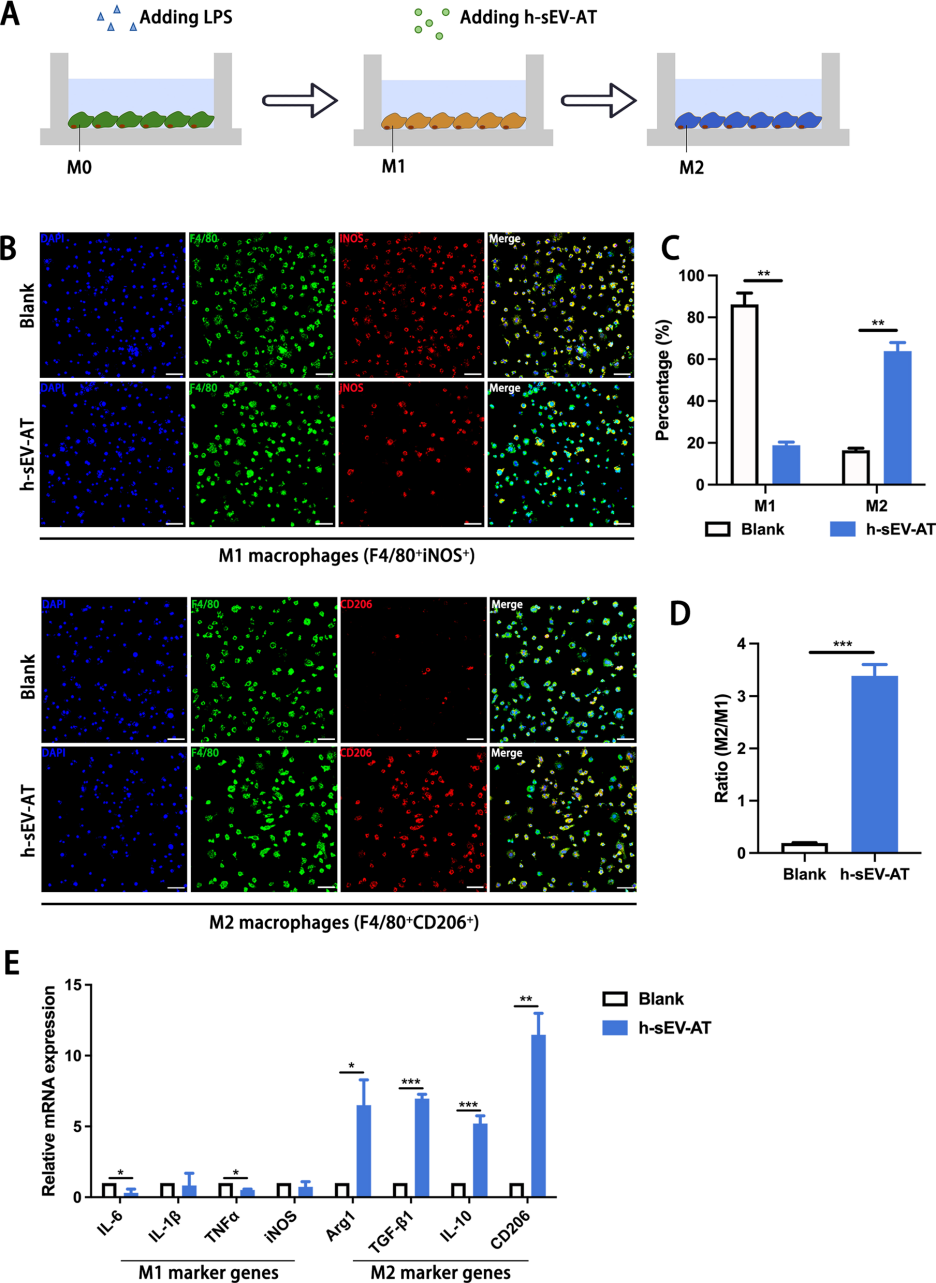
Macrophage polarization induced by sEV-AT. **a** Schematic view of the treatment process. M0 macrophages were induced into M1 macrophages by LPS. Then, the culture medium was removed, and h-sEV-AT was added to polarize M1 macrophages to M2 macrophages for 4 days. **b** Representative images of M1 macrophages (F4/80^+^iNOS^+^) and M2 macrophages (F4/80^+^CD206^+^) with immunofluorescence staining (green: F4/80, red: iNOS/CD206, blue: DAPI). Scale bar = 50 µm. **c** The percentage of M1 macrophages or M2 macrophages in total cells per field of view (scale bar = 50 µm) was analyzed by ImageJ software (*n* = 3). **d** The ratio of M2/M1 per field of view (scale bar = 50 µm) was analyzed (*n* = 3). **e** The expression of M1 macrophage marker genes (IL-6, IL-1β, TNFα, and iNOS) and M2 macrophage marker genes (Arg1, TGF-β1, IL-10, and CD206) was measured by qRT-PCR (*n* = 3). The significance was tested with an unpaired two-tailed Student’s *t* test. *p < 0.05, **p < 0.01, ***p < 0.001

To evaluate the effect of h-sEV-AT on the polarization macrophages, we pre-treated M0 macrophages with 100 ng/ml LPS for 4 h to mimic M1 macrophages, then cultured M1 macrophages with 100 ug/ml h-sEV-AT for 4 days (Fig. 3A). Immunofluorescence staining showed that the percentage of M1 macrophages was decreased and the percentage of M2 macrophages was increased by adding h-sEV-AT. However, in the blank group, most of the cells were M1 macrophages (Fig. 3B-3D). The expression of M1 marker genes (IL-6, IL-1β, TNFα, and iNOS) and M2 maker genes (Arg1, TGF-β1, IL-10, and CD206) was also detected by qRT-PCR. The results showed that the expression of M1 marker genes (IL-6 and TNFα) was decreased and the expression of M2 marker genes (Arg1, TGF-β1, IL-10, and CD206) was significantly increased by h-sEV-AT (Fig. 3E).

### Adipose tissue regeneration induced by h-sEV-AT

**Fig. 4:**
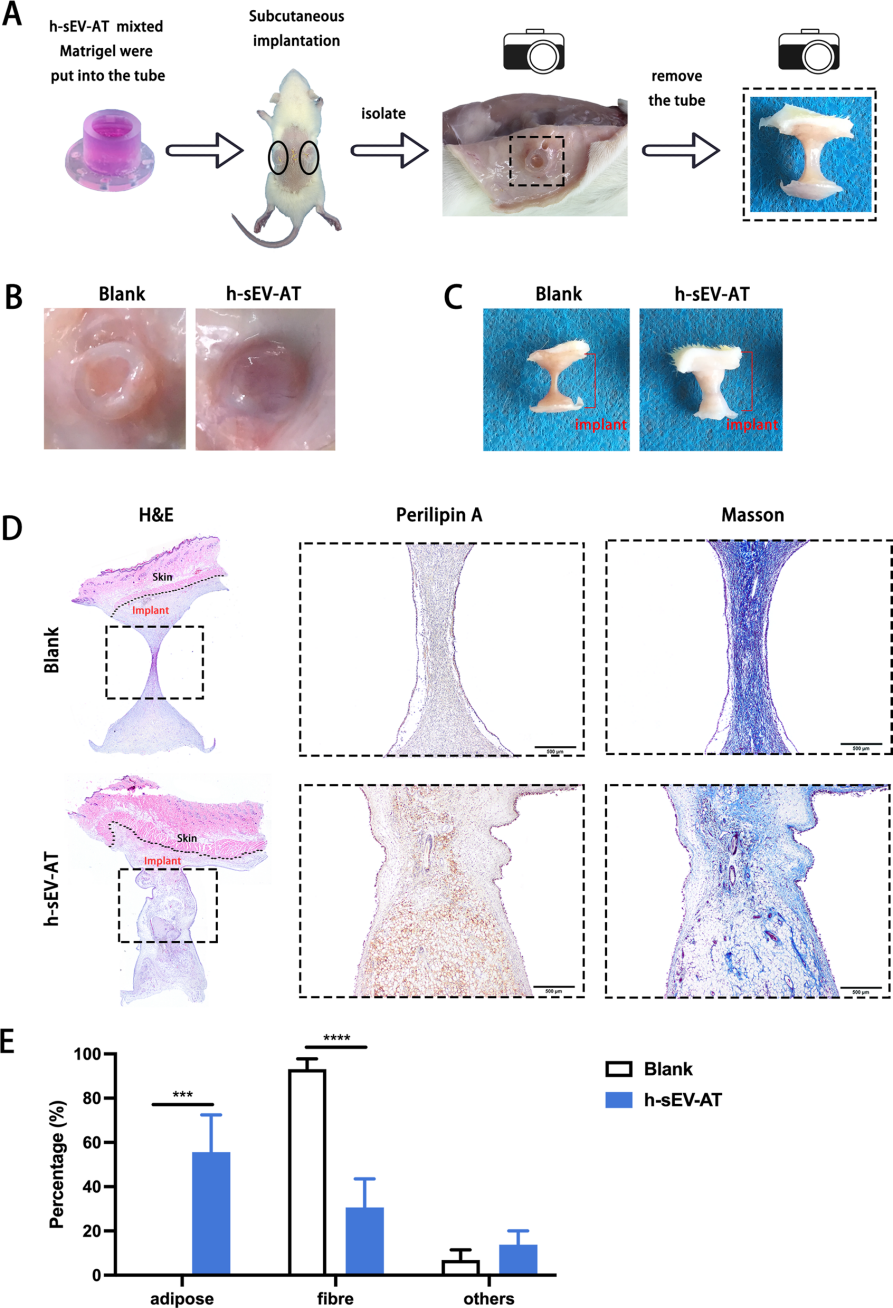
Adipose tissue regeneration induced by h-sEV-AT. **a** Schematic view of the experimental operation process. h-sEV-AT, mixed with Matrigel, was put into the tube and subcutaneously implanted into the back of SD rats. After 4 weeks of implantation, implants with **b** or without **c** the tube were photographed. **d** Representative images of the longitudinal section of the implants at 4 weeks with H&E staining (black dotted square: areas were magnified), immunohistochemically staining (perilipin A), and Masson staining. Scale bar = 500 μm. **e** The percentage of adipose area, fiber area, and other areas in the longitudinal section was analyzed (*n* = 4). The significance was tested with an unpaired two-tailed Student’s *t* test. ***p < 0.001, ****p < 0.0001.

To evaluate the effectiveness of h-sEV-AT in inducing adipose tissue regeneration, h-sEV-AT, mixed with Matrigel, were transferred into the tube and subcutaneously implanted into the back of SD rats (Fig. 4A). After 4 weeks of implantation, the implants with tube were photographed (Fig. 4B). Then, the tubes were removed, and the implants were isolated. Macroimages showed that the volume of regenerated tissue in the h-sEV-AT group was significantly larger than that in the blank group (Fig. 4C). Immunohistochemical staining with adipocyte marker (perilipin A) indicated that a large number of adipocytes could be detected in the h-sEV-AT group, which was surrounded by a small amount of fiber stained by Masson staining. However, in the blank group, no regenerated adipocyte was detected, and nearly all contents were composed of fibers (Fig. 4D and 4E). These results indicated that h-sEV-AT could successfully promote adipose tissue regeneration within 4 weeks.

### Skin wound healing promoted by h-sEV-AT

**Fig. 5:**
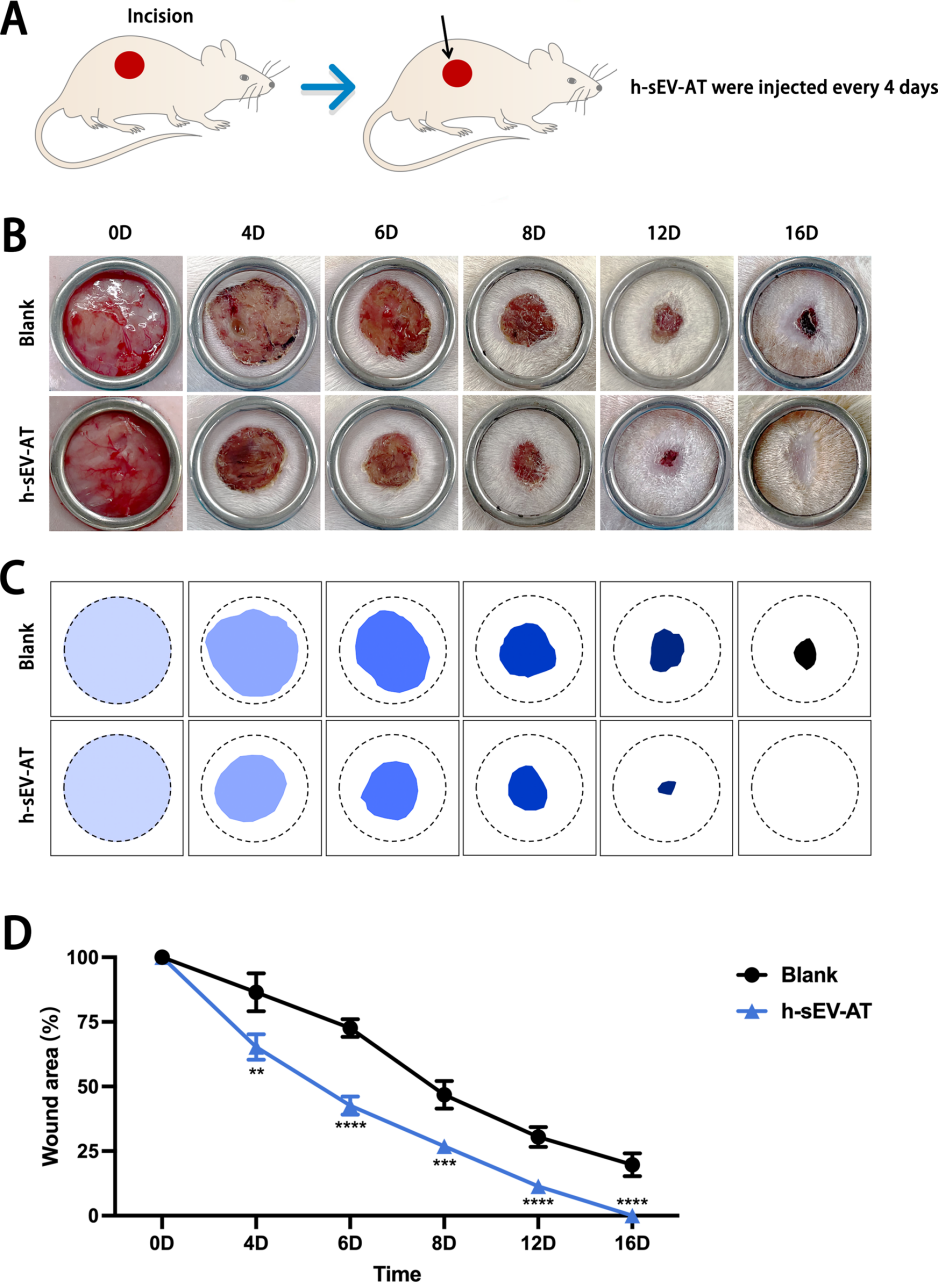
Skin wound healing accelerated by h-sEV-AT. **a** Schematic view of the experimental operation process. Full-thickness skin defects were made, and h-sEV-AT was injected every 4 days. **b** Representative digital photographs of wound areas on day 0, day 4, day 6, day 8, day 12, and day 16. **c** Pattern graphs of wound areas were established for each time point. The dotted circles pointed out the initial wounds, and the colored parts pointed out the wound areas at different time points. **d** The percentage of wound areas in initial wounds was analyzed (*n* = 4). The significance was tested with an unpaired two-tailed Student’s *t* test. **p < 0.01, ***p < 0.001, ****p < 0.0001

**Fig.6:**
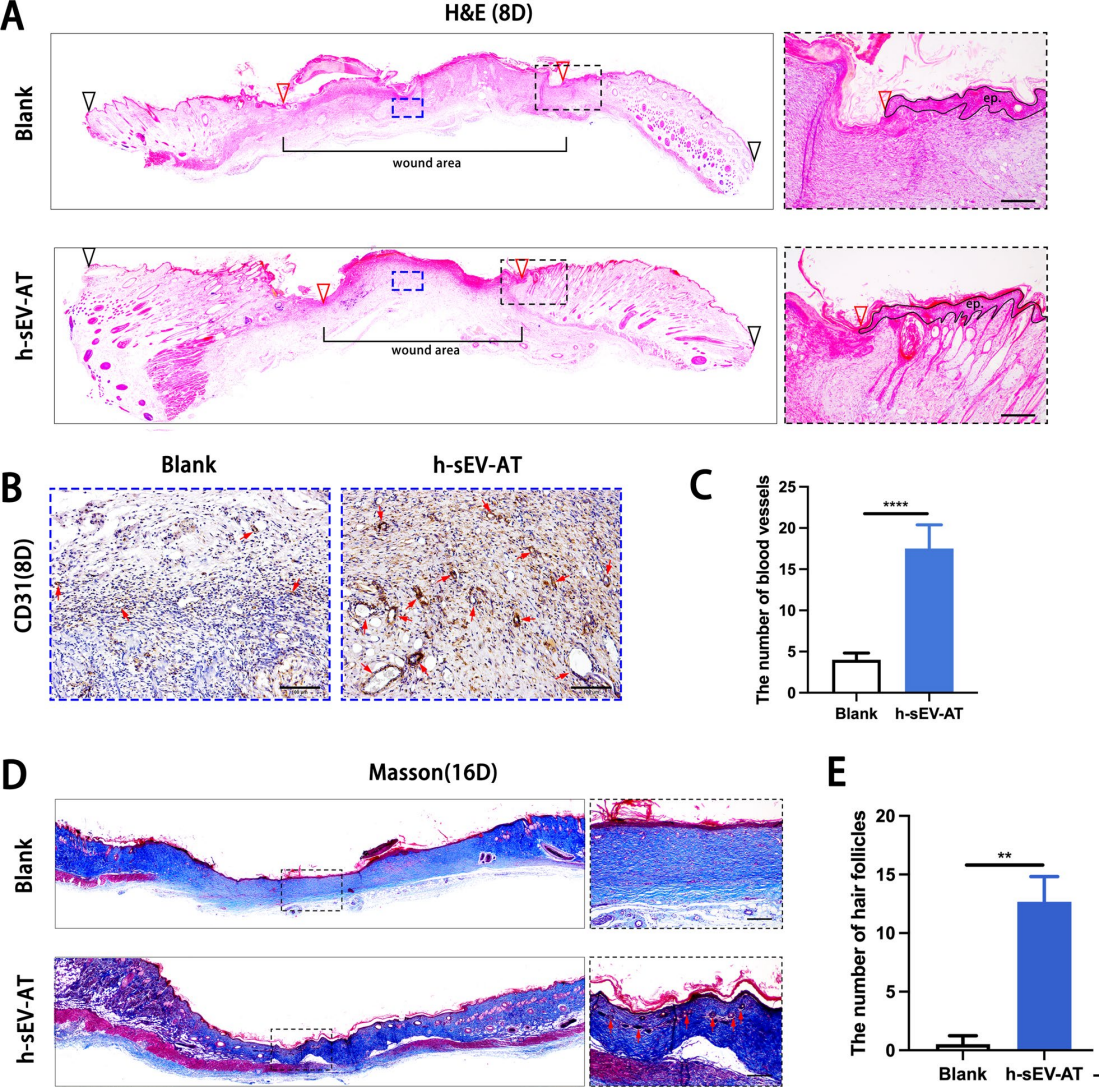
Skin wound healing in microstructure was improved by h-sEV-AT. **a** Representative images of the longitudinal section of the wounds on day 8 with H&E staining. The black inverted triangles pointed out the edge of the initial wound. The red inverted triangles pointed out the edge of wound areas/the edge of re-epithelization. The black dotted boxes represented areas that were magnified. In the right magnified panels, the black lines pointed out the area of the regenerated epithelium (ep.). Scale bar = 100 μm. The blue dotted boxes represented areas with immunohistochemical staining that were magnified in (**b**). **b** Representative CD31-stained images on day 8. The red arrows pointed out typical positive-stained blood vessels. Scale bar = 100 μm. **c** The number of CD31-stained blood vessels per field of view (scale bar = 100 μm) was analyzed (*n* = 4). **d** Representative images of the longitudinal section of the wounds at day 16 with Masson staining. The black dotted boxes represented areas that were magnified. In the right magnified panels, the red arrows pointed out regenerated hair follicles. Scale bar = 100 μm. **e** The number of hair follicles per field of view (scale bar = 100 μm) was analyzed (*n* = 4). The significance was tested with an unpaired two-tailed Student’s *t* test. ****p < 0.0001

To further investigate the ability of h-sEV-AT to repair full-thickness skin defects, we subcutaneously injected h-sEV-AT around the wounds at 4 points (Fig. 5A). Digital photographs were taken on day 0, day 4, day 6, day 8, and day 12 and day 16 to measure the closure rate (Fig. 5B). Pattern graph was made to indicate the speed of wound closure more clearly. The dotted circle represented the scope of the initial wound, and the colored part represented the area of the unhealed wound at different time points (Fig. 5C). By calculating the percentage of the wound area, the results showed that h-sEV-AT could effectively accelerate skin wound healing (Fig. 5D). In microstructure, wounded skins were collected at day 8 followed by H&E staining and immunohistochemical staining and collected at day 16 followed by Masson staining. Compared with the blank group, the re-epithelialization was significantly accelerated by subcutaneous injection of h-sEV-AT. The edge of re-epithelialization was pointed by red inverted triangles (Fig. 6A). Besides, CD31-stained blood vessels were denser and more mature in the h-sEV-AT group than that in the blank group on day 8 (Fig. 6B-6C). Moreover, Masson staining showed that the collagen was irregular in the h-sEV-AT group on day 16. In addition, the regenerated hair follicles indicated by the red arrows could also be observed in the h-sEV-AT group (Fig. 6D-6E). These results indicated that h-sEV-AT could accelerate the healing of full-thickness skin wounds both in the macro- and microstructure.

### Macrophage phenotypes induced by h-sEV-AT in vivo

**Fig. 7:**
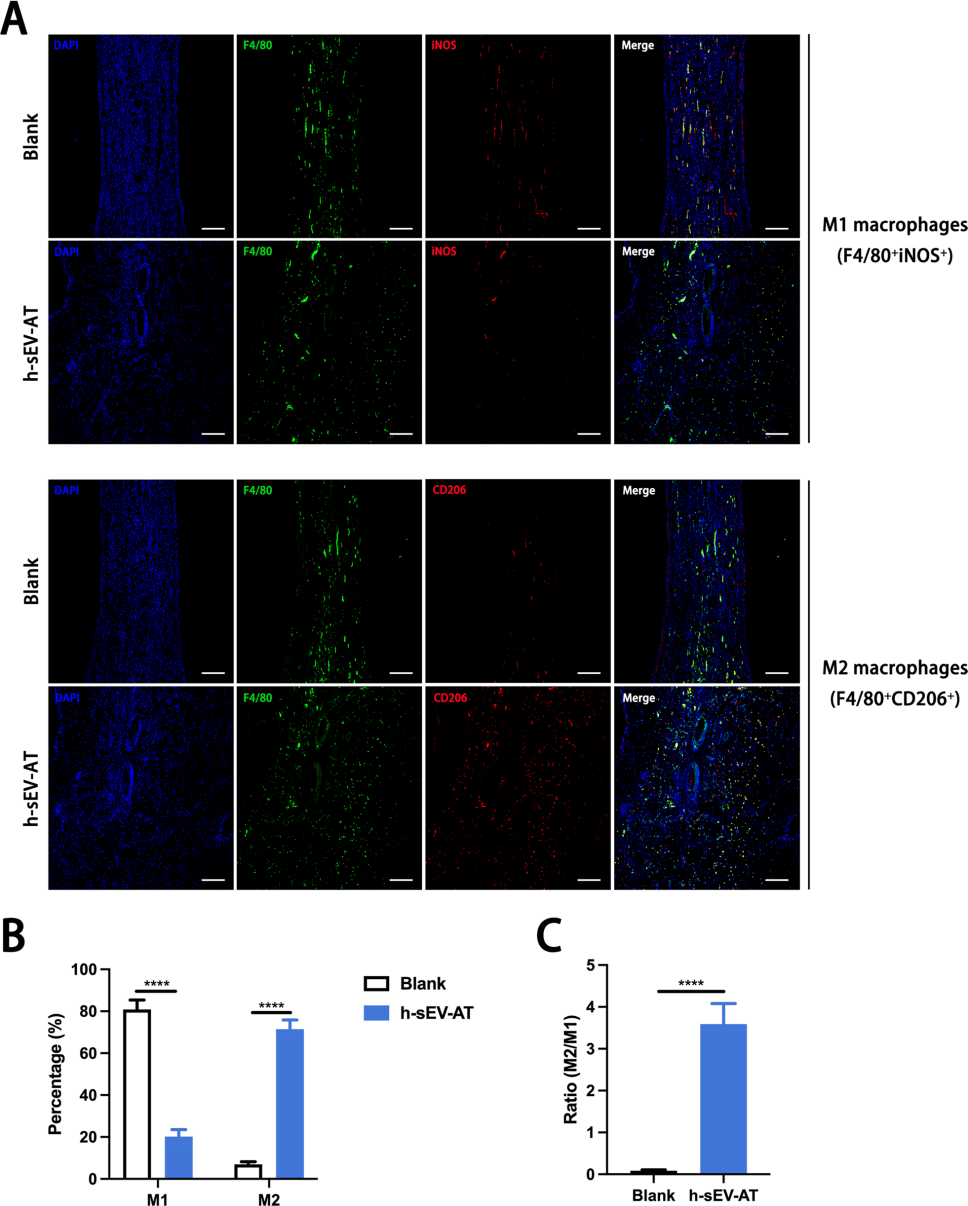
Macrophage phenotypes in adipose tissue regeneration. **a** Representative images of M1 macrophages (F4/80^+^iNOS^+^) and M2 macrophages (F4/80^+^CD206^+^) at 4 weeks with immunofluorescence staining (green: F4/80, red: iNOS/CD206, blue: DAPI). Scale bar = 200 µm. **b** The percentage of M1 macrophages and M2 macrophages in total macrophages per field of view (scale bar = 200 µm) was analyzed by ImageJ software (*n* = 4). **c** The ratio of M2/M1 per field of view (scale bar = 200 µm) was analyzed (*n* = 4). The significance was tested with an unpaired two-tailed Student’s *t* test. ****p < 0.0001

**Fig. 8:**
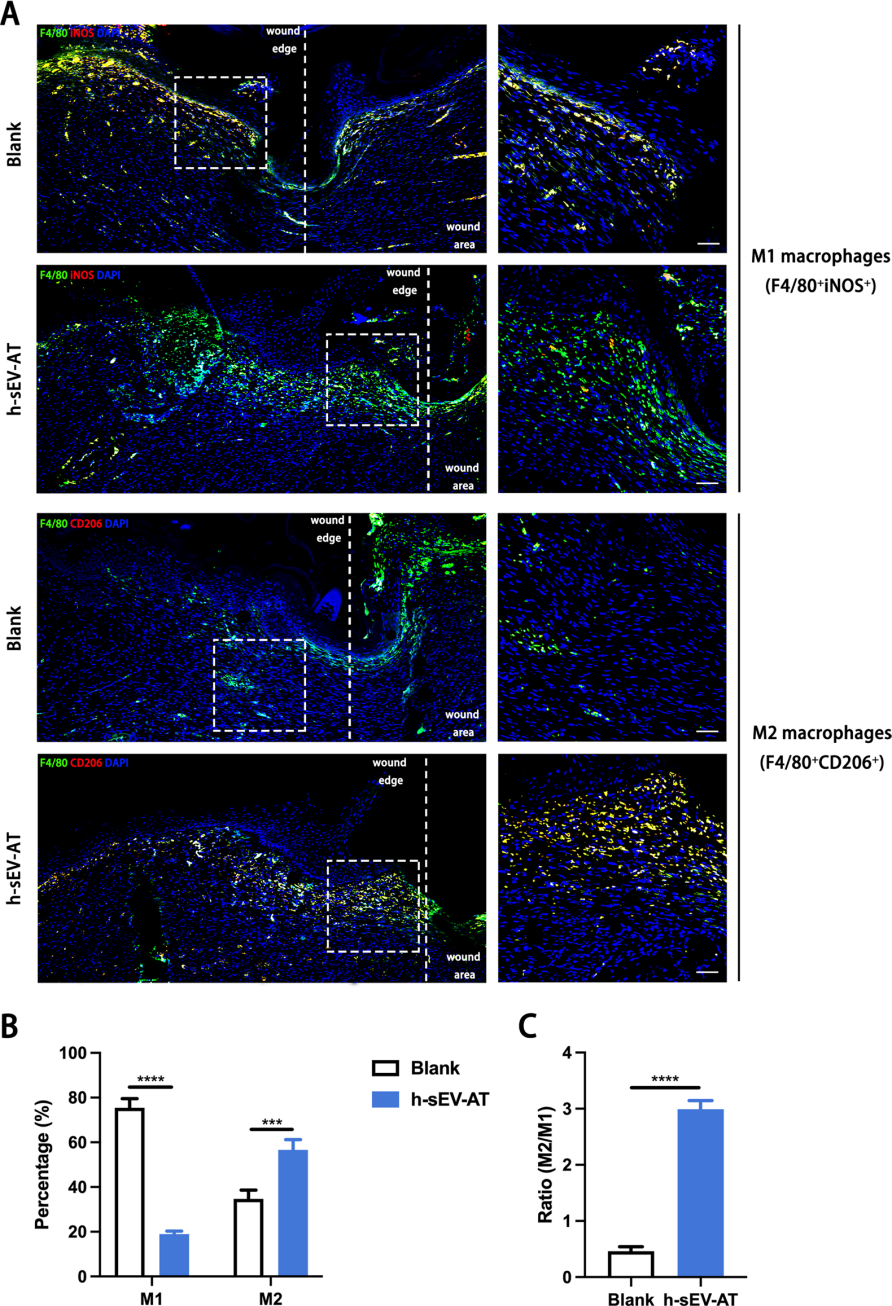
Macrophage phenotypes in skin wound healing. **a** Representative longitudinal section of the wounds at day 8 with immunofluorescence staining (green: F4/80, red: iNOS/CD206, blue: DAPI). The white dotted lines pointed out the edge of wound areas. The white dotted boxes represented areas that were magnified in the right-magnified panels. Scale bar = 50 µm. **b** The percentage of M1 macrophages and M2 macrophages in total macrophages per field of view (scale bar = 50 µm) was analyzed (*n* = 4). **c** The ratio of M2/M1 per field of view (scale bar = 50 µm) was analyzed by ImageJ software (*n* = 4). The significance was tested with an unpaired two-tailed Student’s *t* test. ***p < 0.001, ****p < 0.0001

To further investigate the influence of h-sEV-AT on macrophage phenotypes in vivo, we evaluated the percentage of M1 macrophages and M2 macrophages both in the adipose tissue regeneration and the full-thickness skin wound healing. F4/80^+^iNOS^+^ cells and F4/80^+^CD206^+^ cells were identified as M1 macrophages and M2 macrophages, respectively. For adipose tissue regeneration induced by h-sEV-AT, at 4 weeks, there were more CD206^+^ signals in the h-sEV-AT group but fewer iNOS^+^ signals compared with that in the blank group (Fig. 7A). The percentage of M1 macrophages was only 18.21% but the percentage of M2 macrophages was up to 75.9% in the h-sEV-AT group (Fig. 7B). Besides, we evaluated the ratio of M2/M1 and the result indicated that h-sEV-AT could increase the ratio from 0.19 to 3.76 (Fig. 7C), which had been reported to contribute to tissue regeneration directly [17]. A similar phenomenon also occurred in full-thickness skin wound healing. h-sEV-AT successfully increased the percentage of M2 macrophages and the ratio of M2/M1 at day 8 (Fig. 8). Taken together, h-sEV-AT showed the ability to decrease the percentage of M1 macrophages and increase the percentage of M2 macrophages both in vivo and in vitro, which could cooperate with its role in inducing differentiation of ASCs and ECs and jointly promoted soft tissue repair.

## Discussions

In our previous studies, small extracellular vesicles could be isolated from rat and porcine adipose tissue and these two types of sEVs showed the ability to promote soft tissue regeneration [13]. However, whether small extracellular vesicles could be isolated from human adipose tissue and whether it also showed effectiveness on soft tissue repair were unknown. In this study, we successfully isolated h-sEV-AT from human liposuction adipose tissue and confirmed that h-sEV-AT possessed the characterization of sEVs.

The migration and differentiation of mesenchymal stem cells[18, 19] and endothelial cells[20, 21] were reported to play a crucial role in the regeneration progress. Therefore, we first cultured h-sEV-AT with ASCs and ECs. The results showed that these cells could successfully uptake h-sEV-AT within 6 h. Then, we evaluated the adipogenic differentiation of ASCs and the angiogenic differentiation of ECs induced by h-sEV-AT. The results indicated that h-sEV-AT showed the ability to induce the differentiation of mesenchymal stem cells and endothelial cells. This echoed the previous adipose tissue formation induced by rat and porcine adipose tissue-derived small extracellular vesicles [13], further illustrating that different species-derived sEVs showed similar effects.

The ability of h-sEV-AT to promote adipose tissue regeneration and accelerate skin wound healing was further evaluated. We found that h-sEV-AT could significantly promote adipose tissue formation within 4 weeks. This provided us with a strategy to develop an ideal soft tissue replacement in a short period, which was well-vascularized. For skin wound healing, many types of small extracellular vesicles derived from human cells were used and showed effectiveness. For example, human ASC-derived exosomes were reported to promote skin wound healing [22]. Human umbilical cord mesenchymal stem cells-derived exosomes also accelerated diabetic skin wound healing by increasing the expression of CD31 and Ki69 in the wound area [23]. Human amniotic fluid-derived stem cell-derived exosomes were reported to not only promote skin regeneration but also alleviate scar formation [24]. We noted that some studies pointed out that dermal adipose tissue showed an important function in skin development and repair [25, 26]. However, there was no study focused on the effect of human adipose tissue-derived sEVs on skin wound repair. In this study, by comparison of macro and microstructure, we found that h-sEV-AT could significantly accelerate skin wound healing and effectively induced re-epithelialization, blood vessel formation, and hair follicles regeneration.

The importance of understanding and modulating the inflammatory response was becoming increasingly appreciated for biomaterials in regenerative medicine [27]. Macrophages, a type of important immune cells with great plasticity, had received much attention [11]. Macrophages have been reported to switch phenotypes to modulate inflammation and promote tissue regeneration activities in response to environmental cues [11, 28]. The initial stage is marked by the presence of mostly pro-inflammatory M1 macrophages [29] but lasts for only a short time before giving way to the next phase, in which macrophages with an anti-inflammatory phenotype, like M2 macrophages, which could promote implants integration and tissue regeneration [17, 30–33].

A lot of studies have shown that sEVs possessed the anti-inflammation ability to polarize M1 macrophages to M2 macrophages which facilitated tissue regeneration [34–46]. However, the role of adipose tissue-derived small extracellular vesicles in modulating macrophage phenotypes and whether it was associated with soft tissue repair was unclear. In this study, in vitro, we treated M1 macrophages with h-sEV-AT for 4 days. The results showed that h-sEV-AT could effectively promote the polarization of M1 macrophages to M2 macrophages. In vivo, we also found that the percentage of M2 macrophages was significantly increased by adding h-sEV-AT both in adipose tissue regeneration and skin wound healing.

Different from the sEVs derived from adipose stem cells (sEV-ASCs), there were many other cell types such as adipocytes, endothelial, immune cells, and fibroblasts which contributed to the secretion of sEV-AT. Although tissue-derived sEVs were more heterogeneous, they were closer to the microenvironment under physiological conditions and simpler to isolate than cell-derived sEVs. Besides, the immunoregulatory ability of sEV-AT to polarize macrophages might reflect the low immunogenicity of sEVs, which is beneficial in clinical transformation.

Despite these encouraging results, some limitations of mechanism and clinical transformation need to be further improved. For example, the use of a single component is more conducive to safety evaluation and dose standardization, and more conducive to clinical transformation, so which components of h-sEV-AT played a key role in the repair of soft tissue defects need to be further determined. In addition, M2 macrophages are known to be beneficial to tissue regeneration. However, studies in recent years have shown that the immune regulation mechanism is extremely complex, and all types of macrophages are indispensable in the process of tissue repair [47]. Therefore, it is necessary to further clarify the specific role of different immune cells in the process of tissue regeneration, which is also what clinical transformation of biological agents needs in-depth research.

## Conclusions

In this study, we, for the first study, isolated h-sEV-AT from human liposuction adipose tissue and confirmed that it showed encouraging effectiveness in soft tissue repair. Besides, we also indicated that the ability of sEV-AT to induce soft tissue repair was supported by not only the differentiation of ASCs and ECs but also the polarization of macrophages. Considering the abundant sources, high yield, and guaranteed effectiveness, h-sEV-AT appeared to be a potential raw material for further application.

## Supplementary Information

**Additional file 1: Figure S1**. Extended data for Figure 1. Uncropped Western blot images of (A) Actin, (B) GM130, (C)TSG101, (D) CD81, (E) CD9, and (F) CD63.

## Data Availability

The datasets used and/or analyzed during the current study are available from the corresponding author on reasonable request.

## Abbreviations

h-sEV-AT: Human adipose tissue-derived small extracellular vesicles
ASCs: Adipose-derived stromal/ stem cells
ECs: Endothelial cells
sEV-AT: Adipose tissue-derived small extracellular vesicles
sEVs: Adipose tissue-derived small extracellular vesicles
IL-6: Interleukin 6
IL-1β: Interleukin 1 beta
TNF-α: Tumor necrosis factor-alpha
IL-4: Interleukin 4
IL-13: Interleukin 13
TGF-β1: Transforming growth factor-beta 1
IL-10: Interleukin 10
PPARγ2: Peroxisome proliferator-activated receptor γ2
C/EBPα: CCAAT/enhancer binding protein alpha
FABP4: Fatty acid binding protein 4
VEGF: Vascular endothelial growth factor
FGF2: Fibroblast growth factor 2
