# Supplementary material for: Human adipose tissue-derived small extracellular vesicles promote soft tissue repair through modulating M1-to-M2 polarization of macrophages
Source: Stem Cell Res Ther. 2023 Apr 7;14:67. doi: 10.1186/s13287-023-03306-7 (PMC10080905; doi:10.1186/s13287-023-03306-7)

**Additional file 1**

**Human adipose tissue-derived small extracellular vesicles promote soft tissue repair through modulating M1-to-M2 polarization of macrophages**

Jia Dong^1,2,^ *, Bin Wu^2^, Weidong Tian^1,^*

1. State Key Laboratory of Oral Disease & National Clinical Research Center for Oral Diseases & National Engineering Laboratory for Oral Regenerative Medicine, West China School of Stomatology, Sichuan University, Chengdu, Sichuan, China

2. Department of Stomatology, People's Hospital of Longhua Shenzhen, Shenzhen, Guangdong, China

**Figure S1.** Extended data for Figure 1. Uncropped Western blot images of (A) Actin, (B) GM130, (C)TSG101, (D) CD81, (E) CD9, and (F) CD63.


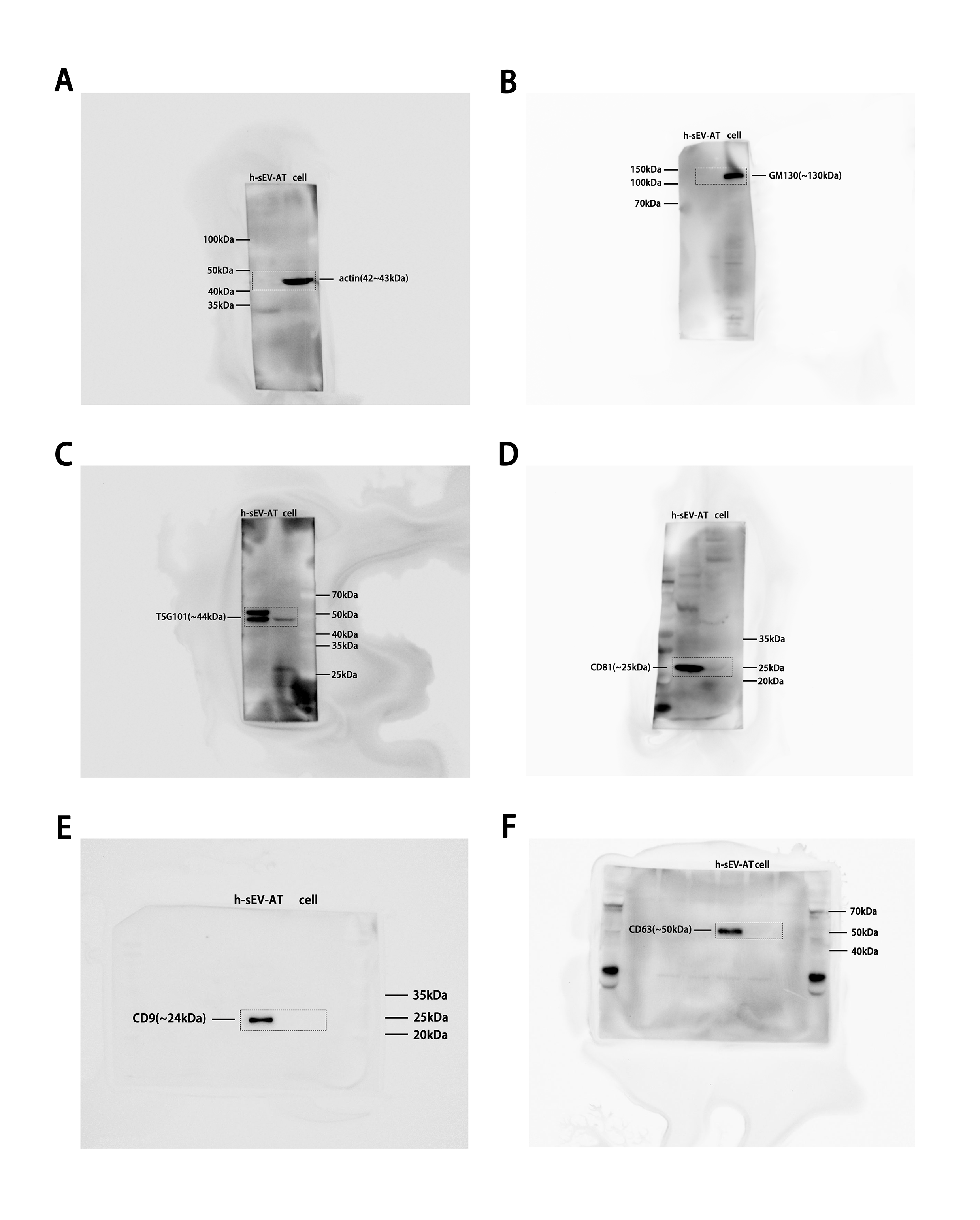

Supplement: Supplementary file 1 — Additional file 1: Figure S1. Extended data for Figure 1. Uncropped Western blot images of (A) Actin, (B) GM130, (C)TSG101, (D) CD81, (E) CD9, and (F) CD63. [file 13287_2023_3306_MOESM1_ESM.docx]
